# COVID-19-related stress and positive coping strategies among young adults in Canada and France: A latent class analysis

**DOI:** 10.1371/journal.pmen.0000261

**Published:** 2025-02-25

**Authors:** Pierre-julien Coulaud, Julie Jesson, Naseeb Bolduc, Emily Jenkins, Chris Richardson, Marie Jauffret-Roustide, Rod Knight

**Affiliations:** 1 Department of Medicine, University of British Columbia, Vancouver, British Columbia, Canada; 2 British Columbia Centre on Substance Use, Vancouver, British Columbia, Canada; 3 École de Santé Publique de l’Université de Montréal, Montréal, Québec, Canada; 4 Centre for Epidemiology and Research in Population Health (CERPOP), Université de Toulouse, Inserm, Université Paul Sabatier, Toulouse, France; 5 School of Nursing, University of British Columbia, Vancouver, British Columbia, Canada; 6 School of Population and Public Health, University of British Columbia, Vancouver, British Columbia, Canada; 7 Centre d’Étude des Mouvements Sociaux (EHESS/CNRS UMR8044/INSERM U1276), Paris, France; 8 Baldy Center on Law and Social Policy, Buffalo University, Buffalo New York, United States of America; 9 Institut Universitaire sur les Dépendances, Montréal, Québec, Canada; 10 Centre de recherche en santé publique (CReSP), Montréal, Québec, Canada; UCL: University College London, UNITED KINGDOM OF GREAT BRITAIN AND NORTHERN IRELAND

## Abstract

Although there is evidence describing how coping strategies can impact mental health outcomes and inequities among young adults, little is known about how different sub-groups of young adults engage with positive coping strategies and the association with mental health challenges. Data were drawn from an online cross-sectional survey (July–December 2021) of young adults aged 18–30 years who reported experiencing COVID-19-related stress in Canada (n=2288) and France (n=1891) during the second year of pandemic. Latent class analysis was used to identify classes with similar coping strategies. Multinomial and logistic regression models were performed in each country to examine differences between these classes in socio-demographic characteristics and mental health. Four classes were identified: high coping (33%), socially engaged (47.1%), self-care and healthy lifestyle (10.1%), and low coping (9.8%). In both countries, young men were more likely to belong to the low coping class and rural residents had an increased likelihood of belonging to the self-care and healthy lifestyle class. Differences between coping classes in socio-demographic characteristics varied by country. In Canada, those who reported financial difficulties were more likely to belong to the self-care and healthy lifestyle and low coping classes, while, in France, descendants of immigrants had increased odds of belonging to the low coping class. Compared to the high coping class, the self-care and healthy lifestyle and low coping classes were more likely to perceive not coping well with stress and reported higher rates of depression, anxiety, and suicidal thoughts. Our findings highlight that specific sub-groups of young adults (men, rural, racialized, economically disadvantaged) may be less likely to engage in positive coping strategies and may experience higher risk of mental health challenges. These findings also underscore the importance of investigating the influence of contextual factors on young adults’ ability to adopt positive coping strategies.

## Introduction

The COVID-19 pandemic and associated public health measures have brought unprecedented sources of stress that have disproportionately affected the mental health and social well-being of young adults (i.e., under 30 years of age) compared to older age groups [1,2]. For example, recent studies in Europe examining age-stratified time trends in mental health demonstrated that young adults experienced higher rates of depressive and anxiety symptoms than older age groups in the initial two years of the COVID-19 pandemic (2020–2022) [3,4]. Previous research has demonstrated how a wide variety of COVID-19-related stressors have impacted young adult mental health, including social- (e.g., loneliness, isolation) [5,6], health- (e.g., getting regularly tested for COVID-19, maintaining social distancing) [7,8], financial- (e.g., job insecurity) and school-related stressors (e.g., moving to online courses) [9–11]. These mental health impacts include symptoms of depression, anxiety and suicidal thoughts. As described in previous studies in North America and Europe [12,13], COVID-19-stressors have significantly disrupted multiple aspects of young adults’ social lives, including their relationships with peers and family, and increased concerns regarding future career opportunities. For instance, a cross-sectional study conducted at five universities in Germany in 2020 found that one in four students experienced a worsened financial situation as a result of the COVID-19-induced economic crisis, and reported higher number of depressive symptoms compared to students with no change/better financial situation [14]. In addition, the context of the pandemic has contributed to exacerbating pre-existing difficulties young adults face in accessing mental health services, especially in times of COVID-19 lockdowns and restrictions (e.g., increased waiting times, barriers to access virtual care) [15]. This has led to high levels of unmet mental health needs, and the need to utilize a range of coping strategies to help mitigate stress within the context of the COVID-19 pandemic [16,17]. Future research is therefore needed to better understand how young adults coped with stress during the COVID-19 pandemic, a time at which both coping mechanisms and mental health well-being were impacted.

Early evidence documented how young adults developed and adopted various coping strategies to mitigate their stress related to the COVID-19 pandemic [18,19]. Informed by previous theoretical frameworks (such as Folkman and Lazarus’s transactional theory of stress and coping [20]), some studies highlighted that young adults who used substances to cope or engaged in avoidance-oriented coping strategies (e.g., ignoring problems by distracting one’s self) had an increased likelihood of experiencing stress and mental health challenges, including depressive and anxiety symptoms [21,22]. While these findings provide important insights on negative coping strategies (e.g., passive, avoidant, and emotion-focused responses), it is essential to identify positive coping strategies that can help young adults meet their mental health needs in the context of highly stressful events, such as the COVID-19 pandemic. For example, some studies found that young adults who connected regularly with family and friends and/or increased physical and outdoor activities reported a lower probability of experiencing stress and other adverse mental health outcomes [23–25]. Research in this area has described how coping strategies can moderate and mediate the relationships between stress and negative mental health outcomes [26,27], underlining the importance of promoting effective coping strategies to prevent adverse mental health experiences. Given that recent longitudinal studies begin to reveal the long-term impacts of the COVID-19 pandemic on the mental health of young adults [10,28], further evidence on positive coping strategies will be critical to inform stress management programs and mental health promotion interventions in the post-pandemic context.

Most of studies examining mental health coping strategies during the pandemic used a variable-oriented approach that focused on the relationships between coping strategies and mental health outcomes separately [29,30]. However, previous researches have demonstrated that various coping strategies can be used simultaneously in beneficial and potentially constitutive ways [31,32]. In order to better account for the heterogeneity of coping patterns, some studies have employed person-centered statistical approaches (such as latent class analysis (LCA)) to identify subgroups with similar combination of coping strategies [33,34]. For example, Hasselle et al., identified four latent profiles (i.e., high and low overall coping, high engagement and disengagement coping) among trauma-exposed young adults [33] whereas Shigeto et al. found six typologies of coping (ranging from “resilient flexible problem-focused copers” to “non-resilient inflexible non-copers”) via an online survey conducted among US young adults [34]. This person-centred approach may refer to the Folkman and Lazarus’ notion of “coping styles” [35], in which individuals may use a combination of strategies to cope more effectively with stressful experiences and mental health challenges.

In addition, emerging data document that the adoption of coping strategies may vary according to socio-demographic characteristics such as age and gender [36]. For example, studies from different settings indicated that women and older groups of students are more likely to engage in positive and creative coping strategies [37,38], while other research has described that some coping strategies (e.g., frequent use of social media, learning something new) had a greater effect on stress and psychosocial well-being among women compared to men [39,40]. Others studies highlighted that sexual and gender minority youth, especially plurisexual (e.g., bisexual, pansexual), transgender, and non-binary youth, reported greater engagement in negative coping strategies (e.g., adverse eating behaviors, self-harm) compared to heterosexuals and cis-gender respondents [41,42]. Another study among North American young adults found direct path between higher income and COVID-19 adaptive coping responses [43]. Significant disparities by ethno-racial status in COVID-19-related coping strategies were also observed among adolescents and students in the US, with lower levels of engaged in positive coping behaviors among ethno-racial minority groups (e.g., Black Latinx, Hispanic) compared to White Americans [44,45].

While a preliminary set of findings showed that young adults adopted various coping strategies during the COVID-19 pandemic, there has been limited researches on examining the different combination of coping strategies that young adults employed to deal with this stressful situation. Moreover, most COVID-19 studies on coping strategies were conducted in a single country, which does not allow opportunities to investigate whether specific contextual factors may influence young adults’ ability to engage in positive coping behaviors. To fill these knowledge gaps, the present study will use data collected from a large and diverse sample of young adults from Canada and France during the second year of the COVID-19 pandemic to respond to the following objectives: 1) identify sub-groups of young adults with a similar coping pattern using latent class analysis; 2) examine whether these coping sub-groups differ by sociodemographic characteristics; and 3) examine the association of sub-group membership and mental health-related outcomes, controlling for sociodemographic characteristics. We hypothesize that our latent analysis will reveal sub-groups of young adults with different patterns of engagement in coping strategies. In addition, coping sub-group membership will vary by young adults’ sociodemographic characteristics. We also hypothesize that sub-group of young adults who will report multiple coping strategies would be less likely to report mental health challenges. Identifying sub-groups of young adults that are engaging in sub-optimal coping strategies is essential to inform equity-oriented mental health promotion and support interventions. Lastly, we anticipate that the distribution of these coping sub-groups and associated sociodemographic factors may vary across our two study contexts. Although Canada and France have some similarities (e.g., both are high-income countries with government-financed health care systems), there are several key contextual and policy differences (e.g., severity and trajectory of COVID-19 cases, availability of mental health resources and programs) that may influence how young adults cope with COVID-19-related stressors [46]. Understanding how these (and other) contextual differences may affect engagement of young adults in positive coping strategies is critically important to inform the development of context-sensitive mental health policies and programs.

## Materials and methods

### Ethics statement

This paper draws on data from the *FOCUS – France Canada Observatory on COVID-19, Youth Health and Social Well-being –* study which is a repeated cross-sectional online research study that explores the social and health experiences of young adults living in Canada and France during the COVID-19 pandemic. All procedures of the FOCUS study comply with the ethical standards of the relevant international and national regulations on human experimentation and with the Helsinki Declaration of 1975, as revised in 2008. This study received ethical approval from the University of British Columbia Behavioural Research Ethics Board (H20-02053). Implied informed consent was obtained from all participants.

### Study design and settings

The first FOCUS survey was conducted from October to December 2020 with a focus on young adult’s experiences with COVID-19 public health measures [47], and their access to mental health services [17]. Given that a significant proportion of FOCUS participants reported not being able to access the mental health services they needed (i.e., one of three young adults in Canada and one of four in France) [17], the second survey was designed to investigate which coping strategies young adults employed in the context of the COVID-19 pandemic. For the present analysis, we used data from the second online survey conducted during the COVID-19 pandemic’s second year, which was launched between July 4 and December 13 2021, a period during which France and Canada were facing the fourth wave of the pandemic.

### Recruitment and data collection procedures

**Fig 1:**
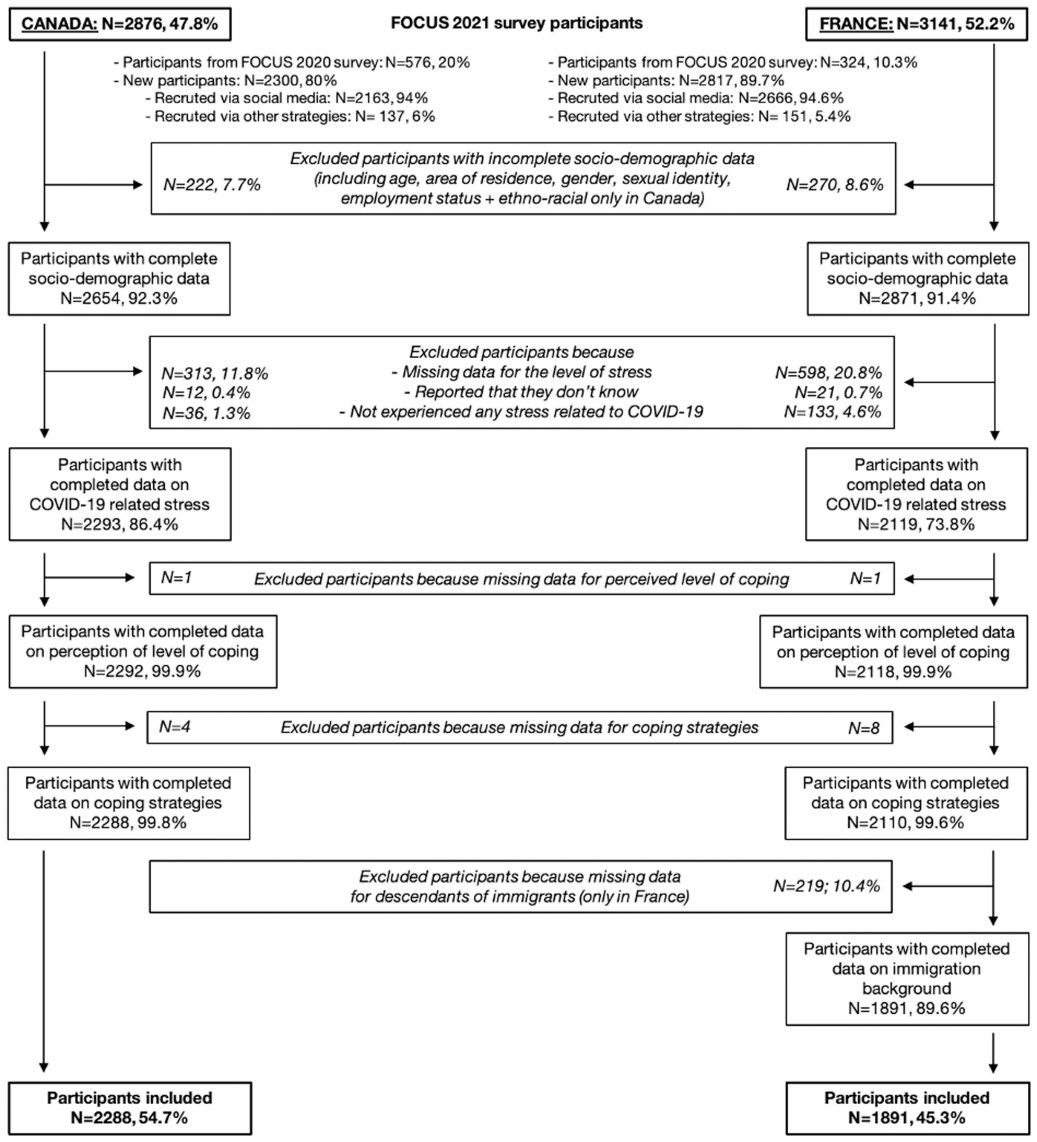
A flow chart of sample selection from the FOCUS 2021 survey. Given that ethno-racial identity is a sensitive and new topic for young adults in the French context, and based on the suggestions of the young adults’ group who conducted the pilot test, the questions about the immigration background (i.e., country of birth of their parents/grand-parents) were asked at the end of the questionnaire in France.

Young adult survey participants were recruited with a convenience sampling approach through two main strategies (see Fig 1). First, an email invitation to participate in the FOCUS 2021 survey was sent to all participants who completed the first online FOCUS survey in Fall 2020 and who provided their consent to be contacted (Canada; n = 1700, France; n = 1235), allowing us to recruit 576 youth in Canada and 324 in France. Second, we promoted the survey through online posts and advertisements on social media (e.g., Facebook, Instagram) and via university partners websites, press articles, and word of mouth to recruit new participants in both countries (Canada; n = 2300, France; n = 2817). To participate in the FOCUS survey, eligible participants were young adults who had reached the age of majority in their jurisdiction of residence (18 or 19 years old, according to the Canadian province or territory, and 18 years old in France) and were not older than 29 years old (30 for those who took part in the first survey); lived in Canada or France; were able to fill out the online questionnaire in English (Canada) or French (either country). The anonymous online questionnaire collected data on socio-demographics, COVID-19 experiences, healthcare access and health outcomes (including mental health) using *Qualtrics*. The online questionnaire was first made in English and then converted to French by two bilingual researchers, one of whom was an English-French translator. The validated mental health scales for assessing depressive and anxiety symptoms (described below), as well as the questions about the coping strategies, were all available in both languages.

In each country, a pre-test questionnaire involving five voluntary young adults was conducted to ensure that the language and wording used in the questionnaire was appropriate for a young adult sample. Details about the study’s objectives and potential risks and benefits were outlined on the first page of the survey. Before they could access the questionnaire, all participants were notified that completing the survey implied informed consent. Participants were able to withdraw from the survey at any time. To ensure the integrity of our online survey and prevent fraudulent submissions (i.e., duplicates, bot infiltrations), we implemented multiple security measures. These included utilizing advanced security features available in our survey platform, Qualtrics, including a Captcha verification question before accessing the survey and detection capabilities to identify false IP addresses, survey duplicates and potential bots. Additional monitoring protocols were also implemented during the data collection period to assess survey completion times and durations and to verify consistency across responses.

### Study population

Our analysis sample included young adults of the FOCUS 2021 survey who had completed the sociodemographic section and questions about their perceived level of coping and use of coping strategies. Participants who indicated that they had not experienced any COVID-19-related stress, and those who had missing information about use of coping strategies were excluded from this analysis (see Fig 1).

### Measures

At the beginning of the coping section, we first questioned participants to express their level of stress in the context of the pandemic: “*Thinking about the amount of stress in your life, would you say that for the last 6 months within the context of COVID-19 your life has felt: not very, a bit, quite or very stressful?*” If participants reported that they did not experience any stress related to COVID-19 they did not complete questions about coping. Because the responses to this question indicated a high prevalence of stress, this was used as a descriptive variable in our analysis.

Second, participants were asked to specify which strategies they used to cope with stress related to COVID-19 in the last 6 months that were effective for managing stress using a multiple-choice question, as follows: *Which of the following have helped you to cope with stress related to the COVID-19 pandemic in the past 6 months?* Participant’s responses included a pre-selected list of 17 coping strategies (e.g., connecting in-person with friends or family, exercising at home, limiting exposure to COVID-19 news), an open-ended question to list other strategies used (these responses were recoded into pre-defined strategies wherever possible), and an option for those who did not use any strategies to cope with stress. Given that the context of the COVID-19 pandemic has placed young adults in specific conditions that have affected their mental health and coping abilities (e.g., lockdown periods) [48], our survey question and list of coping strategies were designed from pre-existing measures used in a large survey assessing the mental health impacts of COVID-19 on the Canadian population [49]. The questionnaire of this Canadian survey was initially designed for a UK longitudinal survey on COVID-19 and mental health in 2020 [50], using a participatory-based approach involving individuals with previous mental health conditions, and was then adapted to reflect the Canadian context through a collaboration with researchers with expertise in mental health (including co-authors CR and EJ) and the Canadian Mental Health Association, a national mental health advocacy organization [49]. This initial list of coping strategies developed by Jenkins et al., 2021 (available here [49]) was then reviewed by a group of volunteers who are young adults via pre-testing of the questionnaire. Based on their feedback, we decided to remove some coping strategies (e.g., connecting with those in my household, accessing government supports) and combine some strategies together (e.g., exercising in my home, doing a hobby, learning or doing something new) to reduce the number of survey responses and facilitate completion of the coping question. The survey questions and list of coping strategies used in the FOCUS survey (both French and English versions) are available in Box A in S1 Text.

Third, participants were asked to assess how well they had coped with COVID-19-related stress using a 4-item scale with the response options: “very well”, “fairly well”, “not very well” to “not well at all”. We dichotomized participants’ responses into two groups to compare those who perceived coping well (“very well” or “fairly well”) and those who did not (“not very well” or “not well at all”). This was used as a first mental health-related outcome to examine the association between our coping classes and the perception of the positive effect of their coping strategies on their level of stress. Three other mental health-related outcomes were included in this analysis. Depressive symptoms were measured using the Patient Health Questionnaire-9 (PHQ-9) [51], which assesses the occurrence and severity of nine depressive symptoms over the past two weeks on a 4-point Likert scale from “not at all” (=0) to “nearly every day” (=3). Total scores range from 0 to 27. Scores of 0–4 indicate minimal, 5–9 mild, 10–14 moderate, 15–19 moderately severe, and 20–27 severe depressive symptoms. To reduce the likelihood of overestimating depression prevalence, a cut-off score of 15 was used to identify participants with major depressive symptoms [52]. The PHQ-9 scale showed good internal consistency in our samples (Canada: α = 0.89, France: α = 0.87). Anxiety symptoms were assessed using the 7-item Generalized Anxiety Disorder scale (GAD-7) [53], which reflects the frequency of symptoms of generalized anxiety in the past two weeks. For each symptom, participant respond on a 4-point Likert scale from “not at all” (=0) to “nearly every day” (=3). As defined in the original instrument, total scores range from 0 to 21, with scores of ≥5, ≥10, and ≥15 indicating mild, moderate, and severe anxiety levels, respectively [53]. In line with the depression scale, we included GAD-7 score as a binary outcome to identify participants with severe anxiety symptoms (cut-off score of 15). The GAD-7 scale demonstrated excellent internal consistency in our study samples (Canada: α = 0.93, France: α = 0.91). Suicidal ideation in the past 6 months was assessed by asking participants: “*Have you considered suicide or taking your own life in the last 6 months?*”.

### Sociodemographic characteristics

We included in this analysis the following characteristics: age, area of residence, gender identity, sexual orientation, ethno-racial identity, living arrangements, and employment status. The age variable was dichotomized based on the median age in our total sample (i.e., 24 years) to differentiate between youth (18–24) and young adults (25–30). Ethno-racial identity data were gathered in different ways in the two countries. In Canada, we used the Canadian Institute for Health Information standards to collect ethno-racial identity [54]. Participants who identified with any ethno-racial identity (one or more) other than white were considered as “ethno-racial minority”. Given that collecting ethno-racial identity data is not allowed in France, we asked participants to provide the birth countries of their parents and grand-parents from both sides to estimate their cultural background. This approach has already been used as a proxy for ethno-racial identity in previous demographic studies conducted in France, including studies on mental health [55,56]. French participants were classified as “descendants of immigrants” if at least one parent or two grand-parents from the same side were born outside Metropolitan France or Europe, according to the French National Institute of Statistics and Economic Studies [57]. To evaluate the impact of the pandemic-induced economic hardship on mental health [9], participants were asked to indicate whether or not the pandemic had impacted their capacity to meet their financial essential needs (e.g., rent, utilities, groceries). Participants who reported a “moderate” or “major impact” were compared with those who selected “none” or “minor impact”. We included these covariates in our analysis based on existing research indicating that these sociodemographic characteristics are associated with coping behaviors and mental health challenges among youth [42,43,49].

### Statistical analysis

First, we conducted an exploratory data analysis to reduce the high number of coping strategies listed in our questionnaire in order to improve interpretability and ensure that the most relevant and meaningful strategies were included. This preliminary step was a constant back-and-forth process between brainstorming sessions with our research team (i.e., inductive analytical approach) and descriptive statistical analysis (i.e., data-driven approach) [58]. We then decided to exclude strategies for accessing mental health and peer support services (i.e., “receiving in-person mental health support”, “virtually connecting with a mental health worker”, “contacting a support group”). While important, these strategies reflect a more complex and dynamic interplay between health services and young adults featuring concepts of help-seeking behaviors, mental health literacy and structural determinants (such as availability of services, administrative and financial barriers) [59]. We also excluded strategies that consisted of “keeping up to date with information” and “increasing use of social media” because they were inversely correlated with “limiting exposure to COVID-19 news” and “limiting exposure to social media”, respectively (see Table A in S1 Text). To guide our selection process of coping variables, we also performed a multiple correspondence analysis (MCA) using the *PROC CORRESP* procedure in SAS on Demand for Academics (SAS Institute Inc.) to graphically represent the relationships between variable categories [60]. In the MCA plot, variables that were positioned close to each other were considered as a similar set of strategies and were then grouped together (see Fig A S1 Text). Specifically, two combined groups of coping strategies were created: self-care and exercising (“maintaining a healthy lifestyle”, “going for a walk/exercise outside”, “exercising in my home/doing a hobby/learning or doing something new”) and community engagement (“having a supportive employer”, “volunteering to help”, “going to local businesses”). Finally, this exploratory analytical approach allowed us to identify six distinct coping strategies that were used in the LCA (see Table 1).

**Table 1:** Description of the set of coping strategies selected after the MCA analysis.

Coping strategies	Survey questionnaire items
Connecting in-person	Connecting in-person with friends or family
Connecting virtually	Connecting with my family or friends virtually (e.g., phone, video chat, etc.)
Self-care and exercising	Maintaining a healthy lifestyle (e.g., balanced diet, enough sleep, exercise, etc.) AND/OR Going for a walk/exercise outside AND/OR Exercising in my home/doing a hobby/learning or doing something new
Limiting exposure to COVID-19 news	Limiting my exposure to the news about COVID-19
Limiting exposure to social media	Limiting exposure to social media (e.g., Facebook, Instagram, Snapchat, Twitter etc.)
Community engagement	Having a supportive employer AND/OR Volunteering to help AND/OR Going to local businesses that are open (e.g., restaurants, hair salons/barber, clothing stores)

Second, we conducted a LCA to identify classes of participants with similar coping strategies using the R package *poLCA* [61] and Mplus version 7.4 [62]. LCA is a widely used statistical procedure to identify qualitatively different subgroups (referred to as latent groups or classes) within populations that share similar characteristics based on observed categorical variables [63]. We tested 2-, 3-, 4-, 5, and 6-class models and allocated participants to the groups for which they had the highest membership probability. The number of latent coping classes was selected based on the overall interpretability and criteria including the Bayesian information criterion (BIC), sample-size adjusted Bayesian information criterion (SABIC), consistent Akaike information criterion (CAIC), and Lo-Mendell-Rubin adjusted (LMR-A) likelihood ratio test. Smaller values for the BIC, SABIC and CAIC indicate a better relative fit [64]. Although not used to select a final model, we also reported the Relative Entropy (RE) to provide additional information on model quality [65]. Furthermore, adequate latent class size (i.e., no less than 5% and no more than 50% of the total sample), parsimony, and the meaningfulness of the combination of coping strategies for each latent class were also considered when selecting the optimal model. To test the reliability of the identified classes, we carried out LCA in the French and Canadian sample separately to assess whether the same classes were found in different samples (see Fig B in S1 Text).

Third, we analyzed the association between sociodemographic characteristics and latent class membership using multinomial logistic regression to investigate predictors of membership in the class of participants that had the most difficulties adopting coping strategies. Lastly, we conducted multivariable logistic regression to examine the association between latent coping classes and mental health outcomes (i.e., perceiving coping well, major depressive symptoms, severe anxiety symptoms, and suicidal ideation in the last 6 months prior the survey) after adjusting for sociodemographic characteristics. These regression analyses were performed using a complete-case approach; participants with missing data for socio-demographics and participants who reported “prefer not to say” for sexual minority status and “other employment status” were excluded. Given that the Canadian and French samples differed significantly in terms of sociodemographic characteristics (see Table 2), all of these regression models were conducted separately using R (version 4.2.3) to identify context-specific differences between young adult’s experiences in Canada and France. This analysis plan was not preregistered.

**Table 2:** Sociodemographic characteristics and coping strategies of young adults in Canada and France, FOCUS 2021 survey (N=4179).

	Canada,	France,	p-value^1^
	N = 2288	N = 1891	
	n (%)	n (%)	
Age (years)			< 0.001
18–24	1144 (50.0)	1047 (55.4)	
25–30	1144 (50.0)	844 (44.6)	
Area of residence			< 0.001
Urban	1,798 (78.6)	1,324 (70.0)	
Rural	490 (21.4)	567 (30.0)	
Gender identity			< 0.001
Woman	1,487 (65.0)	1,243 (65.7)	
Man	531 (23.2)	545 (28.8)	
Other gender^a^	270 (11.8)	103 (5.4)	
Sexual minority^b^			< 0.001
No	1,163 (50.8)	1,297 (68.6)	
Yes	1,083 (47.3)	539 (28.5)	
Prefer not to say	42 (1.8)	55 (2.9)	
Ethno-racial minority (only in Canada)			_
No	1,866 (81.6)		
Yes	422 (18.4)		
Descendants of immigrants (only in France)			_
No		1,186 (62.7)	
Yes		705 (37.3)	
Living with family or friends (missing data; n=12)			< 0.001
No	369 (16.2)	562 (29.8)	
Yes	1,911 (83.8)	1,325 (70.2)	
Employment status			< 0.001
Employed	1,123 (49.1)	801 (42.4)	
Student	955 (41.7)	851 (45.0)	
Unemployed	143 (6.3)	208 (11.0)	
Other employment status	67 (2.9)	31 (1.6)	
COVID-19-related financial impact (missing data; n=2)			0.045
No or minor impact	1,253 (54.8)	1,095 (57.9)	
Moderate or major impact	1,033 (45.2)	796 (42.1)	
Self-reported level of stress			0.8
A bit or not very stressful	653 (28.5)	546 (28.9)	
Very or quite stressful	1,635 (71.5)	1,345 (71.1)	
Coping strategies			
Connecting in-person	1,752 (76.6)	1,451 (76.7)	> 0.9
Connecting virtually	1,280 (55.9)	1,047 (55.4)	0.7
Self-care and exercising	1,835 (80.2)	1,405 (74.3)	< 0.001
Limiting exposure to COVID-19 news	1,249 (54.6)	1,084 (57.3)	0.076
Limiting exposure to social media	805 (35.2)	421 (22.3)	< 0.001
Community engagement	1,341 (58.6)	820 (43.4)	< 0.001

^*1*^ *P-values were calculated from Pearson’s Chi-squared test.*

^*a*^ *Other gender identity includes those who self-identified as non-binary, agender, gender-fluid, gender-queer, or reported another gender identity in an open-text box.*

^*b*^ *Sexual minority includes participants who did not self-identify as straight/heterosexual (including gay/homosexual, lesbian, bisexual, asexual, pansexual, queer, and other sexual orientation with an open-text box).*

## Results

### Participants’ characteristics

Of the 2876 young adults in Canada and 3141 in France who started the FOCUS 2021 survey, more than 90% had complete sociodemographic data in each country (see Fig 1). Of these, we excluded those who did not respond to the question about the level of stress (Canada: n = 313, 11.8%; France: n = 598, 20.8%), those who selected the “I don’t know” response option (Canada: n = 12, 0.4%; France: n = 21, 0.7%), and those who reported having not experienced any stress related to COVID-19 (Canada: n = 36, 1.3%; France: n = 133, 4.6%). These sub-groups of participants excluded were compared to the participants with completed data on COVID-19 related stress (Canada: n = 2293, 86.4%; France: n = 2119, 73.8%) to describe differences in sociodemographic characteristics (see Table B in S1 Text). We then excluded participants who had missing data for perceived level of coping (n = 2) and for coping strategies (n = 12). French participants who did not complete the questions to assess their ethno-cultural origins were then excluded (n = 219, 10.4%). Our analysis sample included 4179 young adults with 2288 (54.7%) residing in Canada and 1891 (45.3%) in France.

As described in Table 2, most participants in both countries were women (Canada: 65%, France: 65.7%), resided in urban areas (Canada: 78.6%, France: 70%), and reported living with family/friends (Canada: 83.8%, France: 70.2%). A higher proportion of participants self-identified as a sexual minority in Canada (47.3%) compared to France (28.5%). In Canada, 18.4% self-identify as an ethno-racial minority, while in France, 37.3% were classified as descendants of immigrants. Regarding the employment status, a higher proportion of unemployed young adults was part of the French sample compared to the Canadian sample (11% versus 6.3%). The vast majority (71% in each setting) reported feeling very stressed and over 40% indicated that the pandemic had a moderate or major impact on their financial obligations in both countries. With regards to coping strategies, more than two-thirds in both settings reported connecting in-person with family/friends (76% in each setting), self-care and exercise (Canada: 80.2%, France: 74.3%), and about half indicated connecting virtually (55% in each setting), limiting exposure to COVID-19 news (Canada: 54.6%, France: 57.3%), and engaging in community activities to cope with COVID-19-related stress (Canada: 58.6%, France: 43.4%). In Canada, one third (35.2%) reported limiting their use of social media while they were 22.3% in France.

### Latent coping classes

**Fig 2:**
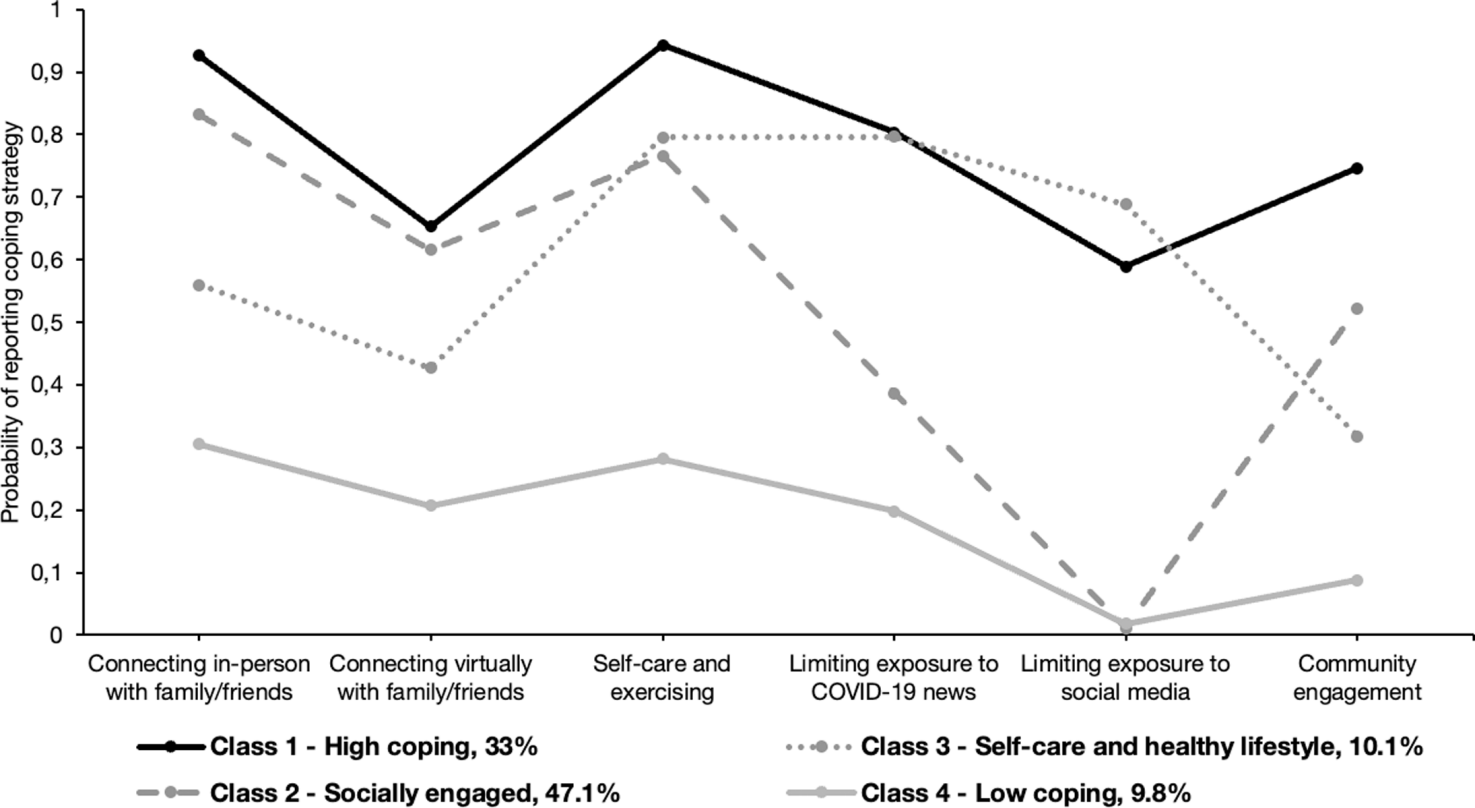
Class-specific probabilities from four-class model of coping strategies, FOCUS 2021 survey (n= 4179).

Table 3 presents the fit statistics for the LCA models. Whilst CAIC indice was slightly better for the three-class model compared to the four-class model, the BIC and SABIC indices showed convergence with the lowest values found for the model with four classes. The results of the LMR-A likelihood ratio test also supported the selection of the four-class model, as adding more classes did not significantly enhance the model fit. Compared to the three-class model, the four class-model identified a specific class of participants (class 3 in Fig 2) who reported moderate social connection and community engagement while using self-care and exercising and limiting exposure to social media and news. This class also provides a more nuanced picture to further explore the role of exposure to COVID-19 news and social media as coping strategies. However, it is important to note that the relative entropy of all models, including the four-class model, was lower than the standard cut-off of 0.6. Overall, given the interpretability of the different latent classes and the values of the information criteria (i.e., BIC, SABIC), which have been considered as the most reliable fit statistics in LCA, we selected the four-class model. When running LCA separately by country, similar classes were also obtained across our study samples (see Fig B in S1 Text).

**Table 3:** Fit statistic for latent class models.

Models	LL	BIC	SABIC	CAIC	RE	LMR-A
2 classes	-15249.39	30607.17	30565.86	30620.17	0.51	
3 classes	-15120.64	30408.04	30344.49	**30428.04**	0.54	**< 0.001**
4 classes	-15089.69	**30404.51**	**30318.72**	30431.51	0.51	**< 0.001**
5 classes	-15079.28	30442.04	30334.00	30476.04	0.51	**0.006**
6 classes	-15072.91	30487.67	30357.39	30528.67	0.52	0.093

*Bold = Ideal class model based on fit statistic; Shaded = Class model selected. LL = log data likelihood; BIC = Bayesian information criterion; SABIC = sample size adjusted BIC; CAIC = Consistent Akaike information criterion; RE = relative entropy; LMR-A = Lo-Mendell-Rubin adjusted likelihood ratio test P-value compared to n-1 number of classes.*

The graphic representation of the class-specific probabilities of reporting coping strategies for the four-class model is presented in Fig 2. Class 1 (n = 1435, 31.6%) was characterized by participants who had the highest probabilities of reporting social connection, self-care and exercise, and community engagement compared to all other classes. Class 1 had high probability of limiting exposure to COVID-19 news and social media, and was labeled the “high coping” class. Class 2 contained the largest latent class (n = 2134, 47%), and was characterized as the “socially engaged” class as it had high probabilities of connecting with family/friends (in-person or virtually) and engaging in community activities. However, half of participants in Class 2 reported limiting exposure to COVID-19 news, and almost none of them reduced their use of social media. Class 3 (n = 503, 11.1%) had high probabilities of self-care and exercise as well as limiting exposure to both news and social media. This was combined with moderate probabilities of endorsing social connection and community engagement. This class was interpreted as the “self-care and healthy lifestyle” class. Finally, Class 4, the smallest group (n = 469, 10.3%) was characterized by participants with lower probabilities of all six coping strategies compared to all other classes and was therefore labeled the “low coping” class.

A statistically significant difference was observed in the distribution of latent coping classes by country (p < 0.001). A greater number of participants in the Canadian sample were in the high coping class compared to the French sample (37.8% versus 27.2%). In France, more than half (52.5%) were classified in the socially engaged class while they were 42.6% in Canada. Similar proportions of participants were found in the self-care and healthy lifestyle class (Canada: 10.7%; France: 9.4%) and in the low coping class (Canada: 8.9%, France: 10.9%) in both countries.

### Association between latent coping classes and sociodemographic characteristics

Tables 4 and 5 show the sociodemographic characteristics of the four latent coping classes and the results of the multinomial logistic regression analysis in each country. Compared to the high coping class, young men had increased odds of belonging to the low coping class (in both countries: Canada: Adjusted Odd Ratio (AOR) [95% Confidence Interval]: 1.63 [1.13–2.35]; France: 2.82 [1.96–4.05]) and the socially engaged class (only in France only: AOR: 1.33 [1.03–1.72]). In addition, living in rural area was associated with an increased likelihood of belonging to the self-care and healthy lifestyle class (in both countries: Canada; AOR: 1.46 [1.02–2.08], France; AOR: 1.58 [1.09–2.28]) and the low coping class (only in Canada; AOR: 1.88 [1.30–2.71]).

**Table 4:** Sociodemographic characteristics associated with latent classes: results of multinomial logistic regression in the Canadian sample (N = 2174).

	High coping class (reference), N = 831	Socially engaged class, N = 938			Self-care and healthy lifestyle class, N = 222			Low coping class, N = 183		
	n (%)	n (%)	AOR	95% CI	n (%)	AOR	95% CI	n (%)	AOR	95% CI
Age (years)										
18–24	391 (47.1)	488 (52.0)	Ref.	—	108 (48.6)	Ref.	—	106 (57.9)	Ref.	—
25–30	440 (52.9)	450 (48.0)	0.89	0.72, 1.09	114 (51.4)	0.99	0.71, 1.37	77 (42.1)	0.72	0.50, 1.03
Area of residence										
Urban	678 (81.6)	747 (79.6)	Ref.	—	166 (74.8)	Ref.	—	127 (69.4)	Ref.	—
Rural	153 (18.4)	191 (20.4)	1.14	0.90, 1.45	56 (25.2)	**1.46**	**1.02, 2.08**	56 (30.6)	**1.88**	**1.30, 2.71**
Gender identity										
Woman	554 (66.7)	632 (67.4)	Ref.	—	133 (59.9)	Ref.	—	110 (60.1)	Ref.	—
Man	179 (21.5)	206 (22.0)	1.02	0.81, 1.29	58 (26.1)	1.28	0.89, 1.82	60 (32.8)	**1.63**	**1.13, 2.35**
Other	98 (11.8)	100 (10.7)	0.86	0.62, 1.18	31 (14.0)	1.41	0.87, 2.29	13 (7.1)	0.65	0.34, 1.23
Sexual minority										
No	432 (52.0)	484 (51.6)	Ref.	—	122 (55.0)	Ref.	—	103 (56.3)	Ref.	—
Yes	399 (48.0)	454 (48.4)	1.02	0.83, 1.25	100 (45.0)	0.81	0.58, 1.14	80 (43.7)	0.92	0.65, 1.30
Ethno-racial minority										
No	677 (81.5)	769 (82.0)	Ref.	—	182 (82.0)	Ref.	—	142 (77.6)	Ref.	—
Yes	154 (18.5)	169 (18.0)	0.94	0.74, 1.20	40 (18.0)	0.94	0.64, 1.39	41 (22.4)	1.23	0.82, 1.83
Living with family or friends										
No	142 (17.1)	147 (15.7)	Ref.	—	36 (16.2)	Ref.	—	24 (13.1)	Ref.	—
Yes	689 (82.9)	791 (84.3)	1.05	0.81, 1.36	186 (83.8)	0.99	0.66, 1.49	159 (86.9)	1.15	0.72, 1.85
Employment status										
Employed	454 (54.6)	452 (48.2)	Ref.	—	110 (49.5)	Ref.	—	82 (44.8)	Ref.	—
Student	334 (40.2)	429 (45.7)	**1.25**	**1.01, 1.54**	92 (41.4)	1.17	0.83, 1.65	86 (47.0)	1.33	0.92, 1.93
Unemployed	43 (5.2)	57 (6.1)	1.33	0.87, 2.03	20 (9.0)	1.78	1.00, 3.20	15 (8.2)	1.71	0.89, 3.28
COVID-19-related financial impact										
No or minor impact	473 (56.9)	540 (57.6)	Ref.	—	106 (47.7)	Ref.	—	86 (47.0)	Ref.	—
Moderate or major impact	358 (43.1)	398 (42.4)	0.96	0.79, 1.16	116 (52.3)	**1.41**	**1.04, 1.90**	97 (53.0)	**1.41**	**1.02, 1.96**

*AOR = Adjusted Odds Ratio; CI = Confidence Interval. Significant associations (p-value < 0.05) are highlighted in bold*.

^*a*^ *Other gender identity includes those who self-identified as non-binary, agender, gender-fluid, gender-queer, or reported another gender identity in an open-text box*.

^*b*^ *Sexual minority includes participants who did not self-identify as straight/heterosexual (including gay/homosexual, lesbian, bisexual, asexual, pansexual, queer, and other sexual orientation with an open-text box)*.

**Table 5:** Sociodemographic characteristics associated with latent classes: results of multinomial logistic regression in the French sample (N = 1803).

	High coping class (reference), N = 484	Socially engaged class, N = 952			Self-care and healthy lifestyle class, N = 173			Low coping class, N = 194		
	n (%)	n (%)	AOR	95% CI	n (%)	AOR	95% CI	n (%)	AOR	95% CI
Age (years)										
18–24	250 (51.7)	537 (56.4)	Ref.	—	102 (59.0)	Ref.	—	112 (57.7)	Ref.	—
25–30	234 (48.3)	415 (43.6)	0.89	0.67, 1.18	71 (41.0)	0.76	0.48, 1.19	82 (42.3)	0.86	0.56, 1.32
Area of residence										
Urban	343 (70.9)	683 (71.7)	Ref.	—	105 (60.7)	Ref.	—	136 (70.1)	Ref.	—
Rural	141 (29.1)	269 (28.3)	0.93	0.73, 1.19	68 (39.3)	**1.58**	**1.09, 2.28**	58 (29.9)	1.03	0.71, 1.51
Gender identity										
Woman	341 (70.5)	629 (66.1)	Ref.	—	113 (65.3)	—	—	97 (50.0)	—	—
Man	114 (23.6)	271 (28.5)	**1.33**	**1.03, 1.72**	51 (29.5)	1.44	0.97, 2.14	88 (45.4)	**2.82**	**1.96, 4.05**
Other	29 (6.0)	52 (5.5)	1.16	0.70, 1.91	9 (5.2)	0.95	0.42, 2.16	9 (4.6)	1.29	0.56, 2.94
Sexual minority										
No	324 (66.9)	689 (72.4)	Ref.	—	116 (67.1)	Ref.	—	143 (73.7)	Ref.	—
Yes	160 (33.1)	263 (27.6)	**0.73**	**0.56, 0.94**	57 (32.9)	1.03	0.69, 1.52	51 (26.3)	0.68	0.46, 1.02
Descendants of immigrants										
No	313 (64.7)	605 (63.6)	Ref.	—	109 (63.0)	Ref.	—	106 (54.6)	Ref.	—
Yes	171 (35.3)	347 (36.4)	1.05	0.83, 1.32	64 (37.0)	1.08	0.75, 1.55	88 (45.4)	**1.57**	**1.11, 2.22**
Living with family or friends										
No	159 (32.9)	280 (29.4)	Ref.	—	43 (24.9)	Ref.	—	67 (34.5)	Ref.	—
Yes	325 (67.1)	672 (70.6)	1.20	0.95, 1.53	130 (75.1)	1.48	0.99, 2.21	127 (65.5)	1.0	0.69, 1.43
Employment status										
Employed	228 (47.1)	403 (42.3)	Ref.	—	78 (45.1)	Ref.	—	77 (39.7)	Ref.	—
Student	204 (42.1)	443 (46.5)	1.19	0.89, 1.61	81 (46.8)	1.00	0.63, 1.59	91 (46.9)	1.32	0.84, 2.08
Unemployed	52 (10.7)	106 (11.1)	1.15	0.78, 1.69	14 (8.1)	0.67	0.34, 1.31	26 (13.4)	1.48	0.84, 2.61
COVID-19-related financial impact										
No or minor impact	283 (58.5)	561 (58.9)	Ref.	—	102 (59.0)	Ref.	—	111 (57.2)	Ref.	—
Moderate or major impact	201 (41.5)	391 (41.1)	0.97	0.78, 1.22	71 (41.0)	1.0	0.69, 1.43	83 (42.8)	1.01	0.71, 1.43

*AOR = Adjusted Odds Ratio; CI = Confidence Interval. Significant associations (p-value < 0.05) are highlighted in bold.*

^*a*^ *Other gender identity includes those who self-identified as non-binary, agender, gender-fluid, gender-queer, or reported another gender identity in an open-text box.*

^*b*^ *Sexual minority includes participants who did not self-identify as straight/heterosexual (including gay/homosexual, lesbian, bisexual, asexual, pansexual, queer, and other sexual orientation with an open-text box).*

Some associations differed by countries. In Canada, reporting a moderate or major financial impact due to COVID-19 was a predictor of membership of the self-care and healthy lifestyle (AOR: 1.41 [1.04–1.90]) and low coping classes (AOR: 1.41 [1.02–1.96]). Student participants (compared to employed youth) had an increased likelihood of belonging to the socially engaged class in Canada (AOR: 1.25 [1.01–1.54). In France, young adults who were classified as descendants of immigrants were more likely to belong to the low coping class (AOR: 1.57 [1.11–2.22]) while those who self-identified as a sexual minority were less likely to belong to the socially engaged class (AOR: 0.76 [0.60–0.96]).

### Association between latent coping classes and mental health outcomes

The results of the logistic regression analyses (Table 6) show that in both countries, participants in the self-care and healthy lifestyle class had higher odds of experiencing major depressive symptoms (Canada: AOR 2.03 [1.45–2.83], France: AOR 1.84 [1.17–2.87]), and reporting suicidal thoughts (Canada: AOR 1.56 [1.13–2.16], France: AOR 1.88 [1.27–2.79]) while they were less likely to perceive coping well with COVID-19-related stress (Canada: AOR 0.65 [0.48–0.88], France: AOR 0.54 [0.38–0.78]). Similar associations were found among participants belonging to the low coping class in Canada. In France, participants in the low coping class had increased odds of reporting major depressive symptoms (AOR: 2.87 [1.89–4.36]) and suicidal thoughts (AOR: 1.60 [1.08–2.34]) than those in the high coping class. No significant association between perceiving coping well and belonging to the low coping class was observed in France. In Canada, young adults in the self-care and healthy lifestyle class were more likely to report severe anxiety symptoms than those in the high coping class (AOR: 1.41 [1.03–1.94]).

**Table 6:** Results of separate multivariate logistic regression analyses examining the association between latent classes membership and mental health outcomes in the Canadian and French samples, FOCUS 2021 survey.

Canada	N = 2174			N = 2104			N = 2150			N = 2173		
	Coping well, N = 1289 (59.3%)			Major depressive symptoms (PHQ-9 > 15), N = 595 (28.3%)			Severe anxiety symptoms (GAD-7 > 15), N = 1050 (48.8%)			Suicide thoughts, N = 707 (32.5%)		
	n (row %)	AOR	95% CI	n (row %)	AOR	95% CI	n (row %)	AOR	95% CI	n (row %)	AOR	95% CI
Latent coping classes												
High coping	518 (62.3)	Ref.		199 (24.5)	Ref.		402 (48.5)	Ref.		260 (31.3)	Ref.	
Socially engaged	573 (61.1)	0.96	0.79, 1.17	235 (26.0)	1.08	0.86, 1.36	427 (46.1)	0.89	0.73, 1.09	276 (29.5)	0.91	0.73, 1.12
Self-care and healthy lifestyle	112 (50.5)	**0.65**	**0.48, 0.88**	88 (40.4)	**2.03**	**1.45, 2.83**	125 (57.3)	**1.41**	**1.03, 1.94**	94 (42.3)	**1.56**	**1.13, 2.16**
Low coping	86 (47.0)	**0.55**	**0.40, 0.77**	73 (42.7)	**2.34**	**1.62, 3.37**	96 (54.5)	1.28	0.91, 1.82	77 (42.1)	**1.54**	**1.08, 2.17**
**France**	**N = 1803**			**N = 1744**			**N = 1769**			**N = 1803**		
	**Coping well, N = 1078 (59.8%)**			**Major depressive symptoms (PHQ-9 > 1 5), N = 332 (19.0%)**			**Severe anxiety symptoms (GAD-7 > 15), N = 614 (34.7%)**			**Suicide thoughts, N = 465 (25.8%)**		
	**n (row %)**	**AOR**	**95% CI**	**n (row %)**	**AOR**	**95% CI**	**n (row %)**	**AOR**	**95% CI**	**n (row %)**	**AOR**	**95% CI**
Latent coping classes												
High coping	294 (60.7)	Ref.		69 (14.7)	Ref.		171 (35.9)	Ref.		116 (24.0)	Ref.	
Socially engaged	596 (62.6)	1.07	0.85, 1.35	164 (17.8)	1.29	0.94, 1.77	308 (32.9)	0.90	0.71, 1.15	223 (23.4)	0.99	0.76, 1.30
Self-care and healthy lifestyle	81 (46.8)	**0.54**	**0.38, 0.78**	40 (23.7)	**1.84**	**1.17, 2.87**	65 (38.0)	1.10	0.75, 1.60	62 (35.8)	**1.88**	**1.27, 2.79**
Low coping	107 (55.2)	0.76	0.54, 1.08	59 (31.7)	**2.87**	**1.89, 4.36**	70 (37.4)	1.16	0.80, 1.68	64 (33.0)	**1.60**	**1.08, 2.34**

*AOR = Adjusted Odds Ratio; CI = Confidence Interval. Significant associations (p < 0.05) are highlighted in bold. All models are adjusted for age, area of residence, gender, sexual orientation, ethno-racial identity, living arrangements, employment status, and COVID-19-related financial impact.*

## Discussion

Globally, the stressors of the COVID-19 pandemic have disproportionately impacted young adults’ mental health, leading to the engagement of a variety of coping strategies to navigate this situation [66]. Our study identified four classes of young adults who used different combinations of coping strategies to manage their stress within the context of the COVID-19 pandemic. A large proportion of young adults who reported feeling stressed because of COVID-19 were classified in the high coping (i.e., all included strategies, 32%) and socially engaged classes (47%). A smaller subset of participants focused on physical wellness (i.e., exercise) and limiting exposure to COVID-19 news and social media (i.e., self-care and healthy lifestyle class, 11%), with the remaining 10% reporting a limited use of any of the coping strategies (i.e., low coping class). The distribution of these latent coping classes differed by country with a greater proportion of participants from Canada in the high coping class while half of the French sample were part of the socially engaged class. In both countries, young men were more likely to belong to the low coping class and rural residents had an increased likelihood of belonging to the self-care and healthy lifestyle class. Our findings also identified how participants in the self-care and healthy lifestyle and low coping classes were more likely to experience adverse mental health outcomes.

Specifically, our findings suggest that being connected with family and/or friends and actively engaged in community activities may represent key strategies for enhancing mental health among young adults during times of public health crisis. As described in previous surveys on coping mechanisms among adolescents and students in various settings [18,39], social connections with family and friends were particularly salient strategies during the COVID-19 pandemic to maintain social interactions, reduce feelings of isolation, and encourage positive thinking and involvement in physical and community activities. While these results tend to suggest social connections and community engagement may have a positive mental health effect, such associations would depend largely on the type, quality and degree of social relationships within young people’s everyday lives [31].

Our findings also suggest the importance of combining coping strategies that enhance social connections. For example, participants in the self-care and healthy lifestyle class who primarily engaged in physical wellness and limited their exposure to social media and COVID-19-related news reported similar adverse mental health outcomes to those in the low coping class. Given we did not assess the motivations of participants for reducing their exposure to media and social media, several hypotheses may explain these findings. This sub-group of participants may have reduced their use of media to avoid stress generated in the media landscape during the pandemic where misinformation and negative news were frequent [67]. Indeed, previous research involving youth has identified associations between pandemic-related news and mental health distress [68,69]. More research is therefore needed to determine how different motivations for use of media and social media may influence young adults’ mental health coping behaviors.

Importantly, our findings highlight that some sub-groups of young adults are not engaging in coping strategies. In both settings, respondents who identified as men were more likely to belong to the low coping class – class that were more likely to experience mental health challenges. Previous COVID-19 research indicates that young men are less inclined to adopt emotional, creative, and supportive coping strategies (such as reaching out to family, starting a new activity, and exercising) compared to women [37,38]. Other research suggests that men report lower frequencies of engaging in multiple coping strategies and instead prefer to focus on task-oriented coping (i.e., taking direct actions in attempt to limit the effects of a stressful situation through cognitive transformations such as planning, positive reframing, sleeping) [70,71]. Similar to previous surveys among university students [72,73], when compared to men, young women in our study reported higher levels of stress (73.7% versus 63.9%), and anxiety symptoms (44.1% versus 33.4%), which may have led to a greater likelihood of women engaging in coping strategies. In addition, geographical location may influence young adults’ ability to engage in protective coping strategies, with those living in rural areas having a higher probability to belong to the self-care and healthy lifestyle class (in both countries) and the low coping class (only in Canada). A previous survey among students in Poland [74] suggested that rural residents may have limited opportunities to connect with others and less access to community activities compared with urban residents for whom social activities and community infrastructures are more widely available.

Individual factors associated with class membership also varied by country. In Canada, young adults who experienced financial difficulties due to COVID-19 were more prone to belong to the self-care and healthy lifestyle and low coping classes. The financial strain induced by the COVID-19-related economic crisis (e.g., lack of employment opportunities, job loss) may have interfered with young adults’ abilities to focus on engaging in coping strategies [75]. Elsewhere and within our previous analyses of FOCUS data, financial support has been identified as a facilitator for coping with COVID-19-related stress, including because financial supports may relieve some of the pressures of needing to find income or employment [19,76]. Compared to the high coping class, students in the Canadian sample were more likely to belong to the socially engaged class, which may be explained by a greater use of social media to stay connected with others and to access information about COVID-19 [77]. In France, we found that those who were classified as descendants of immigrants were more likely to report limited coping strategies. Similar disparities by ethno-racial identity were highlighted in prior research with adolescents and students in the US who reported lower engagement in physical activity and positive coping strategies during the pandemic [32,45].

Compared to their French counterparts, young adults in Canada were more inclined to belong to the high coping class. This difference between France and Canada reveals how contextual factors may influence how young adults differentially adopt coping strategies. First, participants in Canada reported higher rates of depressive and anxiety symptoms, as well as suicidal thoughts, than those in the French sample, suggesting a greater need for young adults in Canada to be supported in the adoption of multiple coping strategies. Second, this difference may reflect a higher level of awareness toward mental health issues in Canada compared to France. For example, two scoping reviews have documented how mental health literacy and promotion research has been prominent in Canada since the year 2000 [78,79], while only a few recent initiatives to promote mental health have been launched in France [80]. Indeed, these disparities feature within sectors other than health; for example, a pre-pandemic study from 2017 reported that 90% of French youth had not received mental health educational programs in their school career [81]. Our own previous FOCUS research has also demonstrated a lower level of mental health-related needs among young adults living in France compared to Canada [17]. Furthermore, the Canadian and French authorities implemented various COVID-19 public health measures during the first two years of the pandemic, which may have differently affected the mental health and coping abilities of young adults in each country. For example, COVID-19 policy responses of the French government were less consistent over time than in Canada with a succession of periods with socially restrictive measures (i.e., lockdown in March-June 2020, series of curfews and lockdowns between October 2020 and April 2021) and periods with less containment measures (e.g., July-October 2020, May-November 2021) [82]. More frequent school and workplace closures were also observed in Canada compared to France during the two first years of the pandemic [82]. In addition, multiple free digital mental health support resources and services (i.e., “Wellness Together Canada” online portal) – including some helplines dedicated specifically for youth – were implemented at the beginning of the pandemic in Canada, while in France, the first mental health initiative (i.e., “Santé Psy Étudiant”; free access to consultations with a psychologist for post-secondary students) was not available until February 2021 [83]. These findings suggest there are important differences in how young adults experience and report mental health-related challenges across our two study contexts.

Our approach has several strengths and limitations. First, we performed our analysis on a convenience sample of young adults, mainly recruited via online postings on social media platforms. Our recruitment strategy may have led to an underestimation of the proportion of participants who reported limiting their exposure to social media in our analysis sample, and therefore influenced the results of our latent analysis. Thus, the resulting sample may not be a representative subset of the young adult population in Canada and France. However, our study sample included a large group of young adults with diverse sociodemographic characteristics (e.g., sexual minority, ethno-racial minority, non-students) that are less often represented in other online surveys. Second, we did not use a validated scale to measure coping strategies which may limit comparability with other research. The phrasing of our coping measure was designed to identify strategies that young adults perceived as effective to cope with stress, which may have influence participants’ selection towards the use of more adaptive strategies than maladaptive coping behaviors. However, the construction of our coping measure was designed with the participation of young adults from diverse backgrounds to develop tailored survey items that reflect relevant coping strategies used by youth themselves during the second year of the COVID-19 pandemic. Our survey also included several measures that are not often investigated, including coping strategies related to use of media and social media – essential resources for young adults to acquire information and enhance social connections during the socially restrictive phases of the pandemic [84]. Given that we did not collect information about coping strategies in the 2020 FOCUS survey, we are unable to assess how coping strategies are changing, including with regards to the social determinants impacting trends over time. Our cross-sectional modelling approach also limits our interpretation regarding possible causal connections between coping classes and mental health outcomes (e.g., reverse causation may explain some of the observed associations). We therefore join others in calling for longitudinal mental health studies of young adults to further examine the social inequities associated with coping strategies and their impact on mental health experiences [85]. Third, we did not collect information about pre-existing mental health conditions, which has been already described as a key determinant for adopting coping behaviors [86].

## Conclusion

Our study enhances our understanding of the different combinations of coping behaviors employed by young adults to manage stress during the second year of the COVID-19 pandemic. Future research using longitudinal data is needed to help inform the development of tailored mental health policies and interventions for young adults. Given the different socio-demographic characteristics of the various profiles across countries, as well as the broader socio-cultural differences related to mental health challenges (e.g., openness to discuss), our study also underscores the importance of investigating the influence of contextual factors on young adults’ ability to adopt positive coping strategies.

## Supporting information

S1 Text Box A in S1 Text – Survey coping measures used in the 2021 FOCUS questionnaire. Fig A in S1 Text – Multiple correspondence analysis (MCA) coordinate plots of coping strategy variables and groups: limiting exposure to social media (“limit_sm”), limiting exposure to COVID-19 news (“limit_news”), connecting in-person with family/friends (red circle: “con in_person”), connecting virtually with family/friends (orange circle: “con_virtually”), self-care and exercising (blue circle: “go_out”, “exerc”, “healthy”), and community support (purple circle: “go_local”, “sup_emp”, “volunt”). Table A in S1 Text – Description of the selected set of coping strategies. Table B in S1 Text – Descriptive comparison of FOCUS survey participants who did not complete the question regarding COVID-19-related stress, those who did not report experiencing stress, and those with completed data on COVID-19 related stress. Fig B in S1 Text – LCA results in the French and Canadian samples.
(DOCX)
