# Supplementary material for: COVID-19-related stress and positive coping strategies among young adults in Canada and France: A latent class analysis
Source: PLOS Ment Health. 2025 Feb 25;2(2):e0000261. doi: 10.1371/journal.pmen.0000261 (PMC12798584; doi:10.1371/journal.pmen.0000261)
Supplement: S1 Text — Fig A in S1 Text – Multiple correspondence analysis (MCA) coordinate plots of coping strategy variables and groups: limiting exposure to social media (“limit_sm”), limiting exposure to COVID-19 news (“limit_news”), connecting in-person with family/friends (red circle: “con in_person”), connecting virtually with family/friends (orange circle: “con_virtually”), self-care and exercising (blue circle: “go_out”, “exerc”, “healthy”), and community support (purple circle: “go_local”, “sup_emp”, “volunt”). Table A in S1 Text – Description of the selected set of coping strategies. Table B in S1 Text – Descriptive comparison of FOCUS survey participants who did not complete the question regarding COVID-19-related stress, those who did not report experiencing stress, and those with completed data on COVID-19 related stress. Fig B in S1 Text – LCA results in the French and Canadian samples. (DOCX) [file pmen.0000261.s001.docx]

Box A. Survey coping measures used in the 2021 FOCUS questionnaire.

English version:

French version:

Fig A. Multiple correspondence analysis (MCA) coordinate plots of coping strategy variables and groups: limiting exposure to social media (“limit_sm”), limiting exposure to COVID-19 news (“limit_news”), connecting in-person with family/friends (red circle: “con in_person”), connecting virtually with family/friends (orange circle: “con_virtually”), self-care and exercising (blue circle: “go_out”, “exerc”, “healthy”), and community support (purple circle: “go_local”, “sup_emp”, “volunt”).

Notes: The coping strategy “Going to local businesses” (go_local) was included in the group *community support* because its meaning was closer to “Volunteering to help” (volunt) and “Having a supportive employer” (sup_emp) than engaging in a physical activities (blue circle).

Table A. Description of the selected set of coping strategies.

| **Self-reported coping strategies with COVID-19-related stress (N=4179)** | **n (%)** | **Inclusion/exclusion** | **Final categories** | **n (%)** |
| --- | --- | --- | --- | --- |
| Connecting in-person with friends or family | 3203 (76.6) | Included | con_inp | 3203 (76.6) |
| Connecting with my family or friends virtually (e.g., phone, video chat, etc.) | 2327 (55.7) | Included | con_virt | 2327 (55.7) |
| Enjoying outdoor activities with friends or family | 2426 (58.1) | Excluded because linked to both family/friends support and going outside |  |  |
| Contacting a support group (i.e., where members with the same issues can come together for sharing coping strategies, to feel more empowered and/or for a sense of community) | 130 (3.1) | Excluded because these strategies required the engagement in health services |  |  |
| Receiving in-person mental health supports | 468 (11.2) |  |  |  |
| Connecting with a mental health worker or counsellor virtually (e.g. via phone, video chat) | 632 (15.1) |  |  |  |
| Spending time with my pet(s) | 1993 (47.7) | Excluded not the focus of the analysis |  |  |
| Maintaining a healthy lifestyle (e.g. balanced diet, enough sleep, exercise, etc.) | 1873 (44.8) | Included and grouped | health_exer | 3240 (77.5) |
| Going for a walk/exercise outside | 2458 (58.8) | Included and grouped |  |  |
| Exercising in my home/doing a hobby/learning or doing something new | 2041 (48.8) | Included and grouped |  |  |
| Limiting my exposure to the news about COVID-19 | 2333 (55.8) | Included | limit_news | 2333 (55.8) |
| Keeping up to date with relevant information (e.g. TV news, newspapers, online information) | 913 (21.8) | Excluded because inversely correlated with limiting exposure to COVID-19 news |  |  |
| Increasing my use of social media (e.g. Facebook, Instagram, Snapchat, Twitter etc.) | 606 (14.5) | Excluded because inversely correlated with limiting exposure to social media |  |  |
| Limiting exposure to social media (e.g. Facebook, Instagram, Snapchat, Twitter etc.) | 1226 (29.3) | Included | limit_sm | 1226 (29.3) |
| Having a supportive employer | 902 (21.6) | Included and grouped | com_sup | 2161 (51.7) |
| Volunteering to help | 485 (11.6) | Included and grouped |  |  |
| Going to local businesses that are open (e.g., restaurants, hair salons/barber, clothing stores) | 1446 (34.6) | Included and grouped |  |  |
| Other strategy, please specify: | 350 (8.4) | Option text responses were recoded with existing options when possible |  |  |
| I don’t know | 58 (1.4) | Excluded |  |  |
| Nothing has helped me to cope with my stress related to COVID-19 | 105 (2.5) | Excluded |  |  |

Table B. Descriptive **comparison of FOCUS survey participants who did not complete the question regarding COVID-19-related stress, those who did not report experiencing stress, and those with completed data on COVID-19 related stress.**

|  | **Canada** | | |  | **France** | | |
| --- | --- | --- | --- | --- | --- | --- | --- |
|  | **Participants with missing data for COVID-19-related stress, N = 313** | **Participants who did not experience COVID-19-related stress, N = 36** | **Participants with completed data on COVID-19 related stress, N = 2293** |  | **Participants with missing data for COVID-19-related stress, N = 598** | **Participants who did not experience COVID-19-related stress, N = 133** | **Participants with completed data on COVID-19 related stress, N = 2119** |
|  | **n (%)** | **n (%)** | **n (%)** |  | **n (%)** | **n (%)** | **n (%)** |
| Age (years) |  |  |  |  |  |  |  |
| 18-24 | 178 (56.9) | 19 (52.8) | 1148 (50.1) |  | 407 (68.1) | 86 (64.7) | 1181 (55.7) |
| 25-30 | 135 (43.1) | 17 (47.2) | 1145 (49.9) |  | 191 (31.9) | 47 (35.3) | 938 (44.3) |
| Area of residence |  |  |  |  |  |  |  |
| Urban | 231 (73.8) | 19 (52.8) | 1801 (78.5) |  | 386 (64.6) | 88 (66.2) | 994 (70.1) |
| Rural | 82 (26.2) | 17 (47.2) | 492 (21.5) |  | 212 (35.5) | 45 (33.8) | 633 (29.9) |
| Gender identity |  |  |  |  |  |  |  |
| Man | 99 (31.6) | 18 (50.0) | 534 (23.3) |  | 234 (39.1) | 70 (52.6) | 625 (29.5) |
| Woman | 182 (58.1) | 14 (38.9) | 1489 (64.9) |  | 349 (58.4) | 57 (42.9) | 1382 (65.2) |
| Other gender^a^ | 32 (10.2) | 4 (11.1) | 270 (11.8) |  | 15 (2.5) | 6 (4.5) | 112 (5.3) |
| Sexual minority^b^ |  |  |  |  |  |  |  |
| No | 180 (57.5) | 26 (72.2) | 1166 (50.9) |  | 484 (80.9) | 107 (80.5) | 1440 (68.0) |
| Yes | 128 (40.9) | 8 (22.2) | 1085 (47.3) |  | 95 (15.9) | 20 (15.0) | 609 (28.7) |
| Prefer not to say | 5 (1.6) | 2 (5.6) | 42 (1.8) |  | 19 (3.2) | 6 (4.5) | 70 (3.3) |
| Q47_employ_tri |  |  |  |  |  |  |  |
| Employed | 177 (56.5) | 23 (63.9) | 1128 (49.2) |  | 248 (41.5) | 50 (37.6) | 888 (41.9) |
| Other | 6 (1.9) | 0 (0.0) | 67 (2.9) |  | 7 (1.2) | 4 (3.0) | 38 (1.8) |
| Student | 115 (36.7) | 11 (30.6) | 955 (41.6) |  | 299 (50.0) | 64 (48.1) | 970 (45.8) |
| Unemployed | 15 (4.8) | 2 (5.6) | 143 (6.2) |  | 44 (7.4) | 15 (11.3) | 223 (10.5) |
| Ethno-racial minority |  |  |  |  |  |  |  |
| White | 263 (84.0) | 31 (86.1) | 1871 (81.6) |  |  |  |  |
| Non-white | 50 (16.0) | 5 (13.9) | 422 (18.4) |  |  |  |  |
| Descendants of immigrants | |  |  |  |  |  |  |
| Yes |  |  |  |  | 2 (0.3) | 43 (32.3) | 707 (33.4) |
| No |  |  |  |  | 0 (0.0) | 68 (51.1) | 1192 (56.3) |
| Missing data |  |  |  |  | 596 (99.7) | 22 (16.5) | 220 (10.4) |

*Notes: ^a^ Other gender identity includes those who self-identified as non-binary, agender, gender-fluid, gender-queer, or reported another gender identity in an open-text box. ^b^ Sexual minority includes participants who did not self-identify as straight/heterosexual (including gay/homosexual, lesbian, bisexual, asexual, pansexual, queer, and other sexual identity with an open-text box).*

Fig B. LCA results in the French and Canadian samples.
